# Blended learning experience, student engagement, and civic engagement in university physical education with ideological and political education

**DOI:** 10.3389/fpsyg.2026.1888349

**Published:** 2026-09-25

**Authors:** Peng Wang, Kangrong Luo

**Affiliations:** Hongyi College, Chengdu Technological University, Chengdu, China

**Keywords:** blended learning experience, civic engagement, community of inquiry, fsQCA, ideological and political education, structural equation modeling, student learning engagement, university physical education

## Abstract

**Objective:**

This study examined how Community of Inquiry (CoI)-based perceived blended learning experience and student learning engagement were associated with civic actions and civic intentions in university physical education incorporating ideological and political education. The study links three forms of learning presence and three dimensions of engagement with behavioral and intentional civic outcome scores in a large multi-site Chinese sample.

**Methods:**

A cross-sectional survey was conducted among 2,035 undergraduate students from 15 universities in Sichuan Province, China. CoI-based perceived blended learning experience was represented by teaching, social, and cognitive presence, and student learning engagement by behavioral, emotional, and cognitive engagement. A 69-item hierarchical structural equation model was estimated using robust maximum likelihood; ordered-indicator WLSMV, Monte Carlo interval estimation, and an equal-loading unmeasured latent method construct were used for sensitivity analyses. Exploratory fuzzy-set qualitative comparative analysis examined configurations associated with high and non-high civic action and intention scores.

**Results:**

Under robust maximum likelihood, perceived blended learning experience was positively associated with student learning engagement (*β* = 0.530), civic actions (*β* = 0.490), and civic intentions (*β* = 0.447), while student learning engagement was positively associated with civic actions (*β* = 0.375) and civic intentions (*β* = 0.396; all *p* < 0.001). Indirect associations through student learning engagement were significant for civic actions (*β* = 0.199) and civic intentions (*β* = 0.210), and the conclusions were unchanged under ordered-indicator WLSMV, Monte Carlo interval estimation, and the admissible method-factor model. Civic action and intention factors showed substantial empirical overlap. Exploratory configurational solutions were threshold-sensitive and largely reflected profiles in which many learning-presence and engagement conditions were jointly high or low.

**Conclusion:**

CoI-based perceived learning experience and student engagement were consistently related to self-reported civic outcomes in this university physical education context. The findings support an associational account centered on coordinated teaching, social, cognitive, and engagement dimensions; the cross-sectional, single-source design does not establish temporal or causal ordering.

## 1 Introduction

At first glance, a university physical education course appears to be a course about the body: movements are practiced, rules are followed, skills are assessed, and physical fitness is improved. Yet this surface view misses a deeper educational possibility. Physical education is also a socially dense learning space in which students cooperate, compete, negotiate rules, take responsibility for collective outcomes, and experience how individual conduct affects others (Zhou and Colomer, 2024). In contemporary Chinese higher education, this makes university physical education a particularly meaningful site for curriculum-based ideological and political education, which calls for the integration of value formation, knowledge transmission, and competence development across the curriculum (Ministry of Education of the People’s Republic of China, 2020; Zhong et al., 2025). Rather than treating civic responsibility only as an abstract curricular aim, physical education can situate responsibility, fairness, cooperation, resilience, and public-mindedness within lived and embodied learning experiences. Prior research has suggested that physical education and school sport may contribute to young people’s physical, social, affective, and cognitive development when appropriate pedagogical conditions are present (Bailey, 2005; Bailey et al., 2009), and recent evidence from Chinese university contexts shows positive associations between physical education courses integrated with civic education and students’ prosocial behavior (Huang et al., 2023).

However, the presence of civic content in a physical education course does not imply that students will experience the course in the same way or report comparable levels of learning involvement and civic engagement. Much existing discussion of curriculum-based ideological and political education in physical education has focused on why such integration is needed or what content should be embedded, while less attention has been paid to the associations among students’ perceived course experience, learning engagement, and civic orientations (Sui et al., 2025; Xu et al., 2025). The empirical gap therefore concerns how these constructs are related within the course context, without assuming that a single-occasion survey can establish a developmental sequence (Jiang and Huang, 2025).

The question is especially relevant in integrated in-class and extracurricular blended learning. University physical education is no longer confined to face-to-face instruction or physical practice on the sports field. Students may encounter course content through classroom teaching, online resources, digital interaction, reflective assignments, extracurricular practice, campus sports events, community activities, and peer collaboration. Meta-analytic evidence indicates that the effectiveness of blended learning is associated with additional learning time, instructional resources, and opportunities for interaction rather than with technology alone (Means et al., 2013). Research in higher education likewise emphasizes the organization of technologies, pedagogical design, and learning activities (Bernard et al., 2014). In the present study, blended learning describes the course context, whereas its measurement focuses on students’ perceived teaching, social, and cognitive presence within that context.

The Community of Inquiry framework provides a useful theoretical language for representing this perceived learning experience. It describes meaningful educational experience through teaching presence, social presence, and cognitive presence (Garrison et al., 2000). Teaching presence concerns how learning is designed, organized, facilitated, and directed; social presence concerns whether learners perceive themselves as connected, recognized, and able to communicate within a learning community; cognitive presence concerns the extent to which learners construct meaning through inquiry, reflection, and application. The CoI instrument was developed for online and blended learning settings (Arbaugh et al., 2008), and a Chinese version has been validated in Chinese higher education (Ma et al., 2017). In the present context, the three dimensions provide a theoretically grounded representation of students’ perceived learning experience, while not constituting a direct audit of objective online–offline integration or physical education course design.

Student learning engagement is a theoretically relevant correlate of both perceived learning experience and civic outcomes. Engagement captures how students behave, feel, and think in learning situations and is commonly represented by behavioral, emotional, and cognitive components (Fredricks et al., 2004). In higher education, engagement has been discussed as an interface between learning environments and student development (Kahu, 2013). In technology-supported settings, active participation, affective connection, and cognitive investment are central to the educational relevance of digital tools and blended designs (Bond et al., 2020; Yu et al., 2025). These dimensions are also pertinent to physical education courses in which students encounter cooperation, rules, responsibility, and community-oriented values.

Civic engagement is a multidimensional educational outcome that includes both reported action and intentional or value-oriented components (Alam et al., 2023). Youth civic development research has emphasized behavioral, cognitive, and socioemotional dimensions (Zaff et al., 2010; Metzger et al., 2018). Zaff et al. (2017) developed measures for college students in mainland China that distinguish Civic Actions (CA) from Civic Intentions (CI). Civic actions, including helping, community service, and responses to social problems, are comparatively distal from a single physical education course and may also reflect prior prosocial dispositions, access to participation opportunities, time, social networks, and institutional support. Examining CA and CI separately preserves the theoretical structure of the instrument while allowing their empirical overlap to be evaluated.

A further challenge is methodological. Structural equation modeling (SEM) is suited to estimating theory-guided average associations and indirect associations among latent constructs. Configurational analysis can examine whether combinations of first-order conditions correspond to high or non-high outcome scores (Wu and Wang, 2022; Chen et al., 2024). The approaches answer different questions and are informative when their outcomes are aligned and the sensitivity of set-theoretic solutions is disclosed. Prior applications combining SEM and fsQCA illustrate this complementary logic (Karuppiah et al., 2024; Akoğul, 2026). The cross-sectional design supports an associational interpretation. SEM therefore serves as the primary analysis, while fsQCA is retained as an exploratory supplementary analysis.

Against this background, the study had three objectives: to examine the associations among CoI-based perceived blended learning experience, student learning engagement, CA, and CI; to estimate the indirect statistical associations between perceived blended learning experience and the two civic outcome scores through student learning engagement; and to explore whether combinations of teaching, social, cognitive, behavioral, and emotional conditions were associated with high and non-high CA and CI. The model integrates learning presence, multidimensional engagement, and civic outcomes in a large multi-site sample from Chinese university physical education. All hypothesized arrows represent theory-guided associations, and the single-occasion design cannot determine temporal sequence.

## 2 Literature review and hypotheses

### 2.1 Blended learning experience in the community of inquiry framework

Blended Learning Experience (BLE) in this study refers specifically to CoI-based perceived learning experience within the focal blended physical education course. The CoI framework represents meaningful learning in online and blended environments through Teaching Presence (TP), Social Presence (SP), and Cognitive Presence (CP; Garrison et al., 2000; Akyol and Garrison, 2008). TP concerns learning design, organization, facilitation, and direction; SP concerns social and emotional connection within a learning community; and CP concerns sustained inquiry, reflection, and meaning construction. The CoI Survey was developed for online and blended learning contexts (Arbaugh et al., 2008), and Ma et al. (2017) supported its application in Chinese higher education. The BLE label is therefore used as shorthand for perceived teaching, social, and cognitive presence rather than as an objective measure of technical integration or overall course quality.

In university physical education incorporating ideological and political education, TP may be associated with clearer understanding of the civic and value-oriented meanings embedded in sport, rules, teamwork, and public responsibility. SP may be associated with belonging, mutual recognition, and cooperative responsibility within the course community. CP may be associated with reflection on the broader social meaning of physical education. The three dimensions were summarized by a higher-order BLE factor while retaining their first-order specificity; alternative measurement models are reported in Supplementary Table S3.

### 2.2 Blended learning experience and civic engagement

Civic engagement is generally understood as intentions and actions directed toward the well-being of communities and broader society. Zaff et al. (2010) argued that engaged citizenship includes behavioral, cognitive, and socioemotional orientations. In the Chinese college student context, Zaff et al. (2017) distinguished Civic Actions (CA) from Civic Intentions (CI). CA and CI capture behavioral and intentional aspects of civic engagement that may differ in their opportunity requirements, even when they are strongly related empirically.

A favorable CoI-based perceived learning experience may be positively associated with both CA and CI. Clear instructional guidance, meaningful peer interaction, and reflective inquiry can coincide with stronger attention to civic values and responsibility. The CA items also refer to general helping, community service, and action on social problems that may occur beyond the focal course; these reports can depend on prior prosocial disposition, participation opportunities, time, resources, and institutional support (Bitton, 2025). The hypotheses therefore concern positive associations rather than course-produced behavior:

*H1a*: Blended Learning Experience (BLE) is positively associated with Civic Actions (CA).

H1b: Blended Learning Experience (BLE) is positively associated with Civic Intentions (CI).

### 2.3 Blended learning experience and student learning engagement

Student Learning Engagement (SLE) is widely regarded as a multidimensional construct involving Behavioral Engagement (BE), Emotional Engagement (EE), and Cognitive Engagement (CE; Fredricks et al., 2004). BE reflects students’ participation, effort, and involvement in learning tasks; EE reflects interest, enjoyment, belonging, and affective connection; and CE reflects deep processing, self-regulation, reflection, and strategic learning. In higher education, engagement is often understood as a bridge between learning environments and student outcomes, because students’ development depends not only on the availability of educational resources but also on the extent to which they invest physical, psychological, and intellectual energy in learning activities (Astin, 1984; Kahu, 2013).

The USEI was developed to measure university students’ engagement across behavioral, emotional, and cognitive dimensions (Maroco et al., 2016). Its Chinese version was validated among Chinese university students by She et al. (2023), supporting the three-dimensional structure and the applicability of the instrument in Chinese higher education settings. These studies provide measurement support for treating SLE as a second-order construct composed of BE, EE, and CE.

Theoretically, BLE should be positively associated with SLE. Strong TP can coincide with clear learning goals, structured tasks, and useful feedback, all of which are relevant to behavioral participation and cognitive effort. Strong SP can coincide with belonging and emotional connection. Strong CP can coincide with inquiry, reflection, and meaning-making. Accordingly, this study proposes:

*H2*: Blended Learning Experience (BLE) is positively associated with Student Learning Engagement (SLE).

### 2.4 Student learning engagement and civic engagement

SLE may also be positively associated with civic engagement. Engagement theory links student outcomes with the quality and quantity of involvement in educational experiences (Astin, 1984). Behavioral engagement reflects participation in course tasks and group activities; emotional engagement reflects attachment to the learning community and its values; cognitive engagement reflects reflection on the social implications of course content and the connection between personal learning and public responsibility.

These associations are relevant to physical education incorporating ideological and political education because cooperation, fairness, discipline, resilience, and collective responsibility are encountered within both physical practice and reflective course activities. Active participation, affective connection, and cognitive investment provide theoretical reasons to expect positive covariation with CA and CI. At the same time, a cross-sectional survey cannot determine whether learning engagement precedes civic orientations or whether civically oriented students engage more strongly in the course. The following hypotheses are proposed:

*H3a*: Student Learning Engagement (SLE) is positively associated with Civic Actions (CA).

*H3b*: Student Learning Engagement (SLE) is positively associated with Civic Intentions (CI).

### 2.5 Indirect associations through student learning engagement

BLE and SLE are expected to show positive associations with civic outcome scores. A corresponding indirect association is present when BLE is related to SLE and SLE is related to CA or CI within the same theory-guided model. Kahu’s (2013) framework likewise positions engagement at the interface between structural and psychosocial features of the learning environment and student outcomes. In cross-sectional data, this specification is an associational decomposition and does not establish mediation over time.

BLE represents students’ perceptions of teaching, social, and cognitive presence; SLE represents their behavioral, emotional, and cognitive involvement; and CA and CI represent self-reported civic outcome scores. Joint modeling tests whether the association between BLE and each civic outcome includes a statistically identifiable indirect component through SLE, without assuming a developmental sequence. Accordingly, the following hypotheses are proposed:

*H4a*: Blended Learning Experience (BLE) has a positive indirect association with Civic Actions (CA) through Student Learning Engagement (SLE).

*H4b*: Blended Learning Experience (BLE) has a positive indirect association with Civic Intentions (CI) through Student Learning Engagement (SLE).

### 2.6 Exploratory configurational perspective

The preceding hypotheses concern average associations among higher-order constructs. Similar civic outcome scores may also correspond to different combinations of first-order learning-presence and engagement conditions. Fuzzy-set qualitative comparative analysis can summarize set-theoretic combinations associated with an outcome and its negation (Ragin, 2008; Fiss, 2011; Woodside, 2013; Muddiman, 2020). Calibration, truth-table thresholds, and correlations among conditions can materially shape the solutions, so the analysis is interpreted as a sensitivity-tested description of associational profiles.

TP, SP, and CP represent distinct forms of perceived learning presence, while BE, EE, and CE represent distinct forms of student engagement. The exploratory configurational analysis aligns these first-order conditions separately with CA and CI and examines both high and non-high outcome sets. This supplementary analysis complements the primary SEM by describing conjunctural patterns while preserving the same outcome definitions.

*RQ1*: Which configurations of Teaching Presence (TP), Social Presence (SP), Cognitive Presence (CP), Behavioral Engagement (BE), Emotional Engagement (EE), and Cognitive Engagement (CE) are associated with high and non-high Civic Actions (CA) and Civic Intentions (CI), and how stable are those configurations across prespecified analytical specifications?

### 2.7 Hypothesized model

Based on the above discussion, the hypothesized model treats BLE as a higher-order construct reflected by TP, SP, and CP; SLE as a higher-order construct reflected by BE, EE, and CE; and CA and CI as civic outcome factors. The structural component estimates the associations of BLE and SLE with CA and CI and the indirect associations from BLE through SLE (Figure 1). First-order configurational patterns are examined separately as an exploratory supplementary analysis.

**Figure 1:**
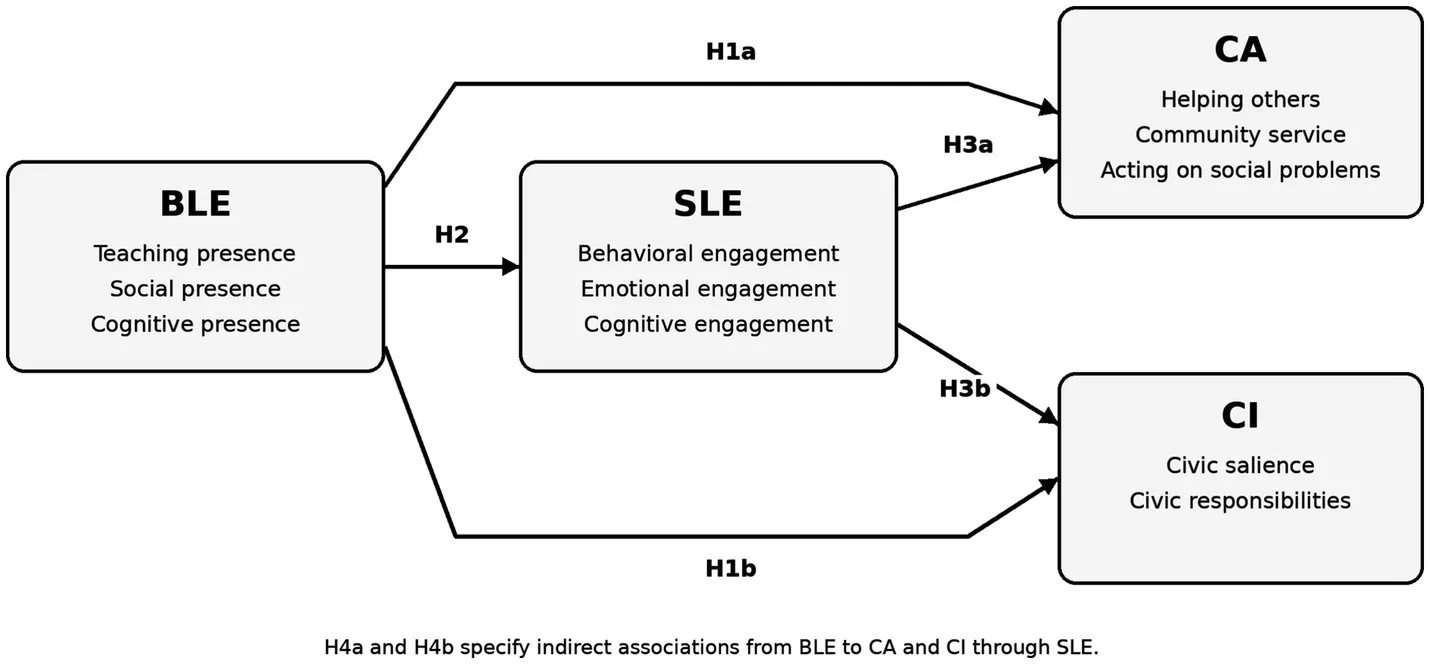
Hypothesized associational model. BLE, blended learning experience; TP, teaching presence; SP, social presence; CP, cognitive presence; SLE, student learning engagement; BE, behavioral engagement; EE, emotional engagement; CE, cognitive engagement; CA, civic actions; CI, civic intentions. Single-headed arrows indicate theory-guided associations. H4a and H4b refer to indirect associations through SLE. The model does not imply temporal or causal ordering.

## 3 Methods

### 3.1 Participants and procedure

This study adopted a cross-sectional, multi-site survey design to examine the relationships among blended learning experience, student learning engagement, and civic engagement in university physical education courses incorporating ideological and political education. Participants were undergraduate students recruited from 15 universities in Sichuan Province, China, including Sichuan University, the University of Electronic Science and Technology of China, Southwestern University of Finance and Economics, Southwest Jiaotong University, Southwest Petroleum University, Chengdu Technological University, and other participating universities. The target population was college students who had participated in university physical education courses that integrated ideological and political education through in-class instruction, online learning activities, and/or extracurricular learning practices.

The inclusion criteria were as follows: participants had to be undergraduate students enrolled at one of the participating universities, aged 18 years or above, and had experience with the focal type of university physical education course. Students who were younger than 18 years old were not invited to participate, and no data from minors were collected. Participation was voluntary, and students could discontinue the survey at any time before submission without any penalty.

Data were collected from October 11 to November 21, 2025. The survey was administered online through Wenjuanxing, a widely used Chinese online questionnaire platform. The questionnaire link was distributed through course-related online communication channels, such as class groups and learning-management communication groups, with the assistance of physical education instructors and course coordinators. To reduce potential response pressure, participants were informed that the survey was for academic research only, that their participation or non-participation would not affect their course grades or evaluations, and that all responses would be analyzed in aggregate form.

Before entering the formal questionnaire, participants were required to read an informed-consent statement on the first page. The statement explained the purpose of the study, eligibility requirements, the voluntary nature of participation, the approximate time required to complete the survey, confidentiality arrangements, and the right to withdraw before submission. Only participants who confirmed that they were at least 18 years old and clicked “I agree” on the informed-consent page were allowed to proceed to the questionnaire. Thus, written informed consent was obtained electronically from all participants prior to data collection. The study protocol was reviewed and approved by the Ethics Committee of the Department of Physical Education, Chengdu Technological University, China. The ethics approval number was CDTU2025TY016.

The online questionnaire was configured to improve data completeness and response quality. All substantive items were set as mandatory in the Wenjuanxing system; therefore, questionnaires with missing values on scale items were not generated. In addition, the relevant questionnaire items were set as single-response items, so invalid cases due to multiple selections were not applicable. The first part of the questionnaire collected demographic and background information, followed by measures of blended learning experience, student learning engagement, and civic engagement. To ensure that participants read the items carefully, one attention-check item was embedded in the questionnaire: “This item is used to check whether you are reading carefully. Please select ‘Agree’ for this statement.” Responses that failed this attention-check item were treated as invalid.

A total of 2,209 questionnaires were returned. Before formal statistical analysis, the data were screened according to prespecified validity criteria. Questionnaires were excluded if they met one or more of the following conditions: (a) the respondent did not meet the eligibility criteria, such as not being an undergraduate student in the target course context or not confirming adult status; (b) the questionnaire was completed in less than 180 s, which was considered an unrealistically short completion time relative to the length and cognitive demands of the survey; (c) the response showed straight-line answering or near-zero response variation across long blocks of scale items; (d) the response showed highly mechanical or patterned answering, such as repeated alternating response sequences across multiple sections; (e) the respondent failed the embedded attention-check item; or (f) duplicate submissions were identified based on overlapping device, browser, IP-related, timestamp, and response-pattern information, in which case only the earliest valid response was retained. Because some responses could meet more than one exclusion criterion, invalid questionnaires were removed at the case level rather than by summing mutually exclusive exclusion categories.

After data screening, 174 invalid questionnaires were removed, resulting in 2,035 valid responses. The effective valid-response rate was therefore 92.12%. The final sample contained 989 male students and 1,046 female students, accounting for 48.60 and 51.40% of the sample, respectively. All participants were adults, with 312 students aged 18 years, 643 aged 19 years, 581 aged 20 years, 337 aged 21 years, and 162 aged 22 years or above. The sample included students from different years of study, academic disciplines, places of origin, ethnicity groups, extracurricular physical activity frequencies, and levels of prior blended learning experience. The demographic and background characteristics of the participants are presented in Table 1.

**Table 1:** Demographic and background characteristics of the participants (*N* = 2,035).

Variable	Category	*n*	%
Gender	Male	989	48.60
	Female	1,046	51.40
Age group	18 years	312	15.33
	19 years	643	31.60
	20 years	581	28.55
	21 years	337	16.56
	22 years or above	162	7.96
Year of study	Freshman	743	36.51
	Sophomore	692	34.00
	Junior	386	18.97
	Senior	214	10.52
Academic discipline	Science and engineering	672	33.02
	Humanities and social sciences	469	23.05
	Economics and management	407	20.00
	Education, arts, and sport	316	15.53
	Medicine and other disciplines	171	8.40
Place of origin	Urban area	746	36.66
	County/town	615	30.22
	Rural area	674	33.12
Only-child status	Only child	904	44.42
	Non-only child	1,131	55.58
Ethnicity	Han ethnicity	1,876	92.19
	Ethnic minority	159	7.81
Weekly extracurricular physical activity frequency	0–1 time/week	394	19.36
	2–3 times/week	947	46.54
	4 or more times/week	694	34.10
Prior blended learning experience	No/limited experience	523	25.70
	Moderate experience	1,026	50.42
	Rich experience	486	23.88

The adequacy of the final sample size was evaluated in relation to the primary SEM. The final sample of 2,035 substantially exceeded common minimum recommendations for structural equation modeling with multiple latent constructs and indirect paths. A conservative *a priori* power approximation based on multiple regression assumed a small effect size of *f*^2^ = 0.02, *α* = 0.05, power = 0.95, and up to 10 predictors, yielding a minimum sample of approximately 1,229. The available sample therefore provided sufficient power for the hypothesized structural associations. The configurational analysis was treated as exploratory and evaluated through truth-table frequency requirements and extensive threshold sensitivity rather than through a separate power calculation.

### 3.2 Measurements

All constructs were measured using established scales developed for Chinese college students or validated in Chinese higher education contexts. The survey instructions and item wording were contextualized to the focal university physical education course and its in-class, online, and extracurricular learning activities. Unless otherwise noted, items were rated on a five-point scale, with higher scores indicating higher levels of the corresponding construct. BLE and SLE were operationalized as higher-order constructs, while civic engagement was represented by CA and CI. The auxiliary overall civic engagement score was calculated as the equal-weighted mean of the two civic scores, CEng = (CA + CI)/2.

Blended Learning Experience (BLE) was measured using the Community of Inquiry Survey Instrument (Arbaugh et al., 2008), whose Chinese version was validated by Ma et al. (2017). BLE was treated as shorthand for CoI-based perceived blended learning experience in the focal course and was represented by Teaching Presence (TP; 13 items), Social Presence (SP; 8 items), and Cognitive Presence (CP; 12 items). Representative items were “The instructor clearly communicated the important topics of this course” for TP, “Interacting with other course participants gave me a sense of belonging” for SP, and “The problems raised in the course increased my interest in the course issues” for CP. The scale captures perceived teaching, social, and cognitive presence; it does not directly observe the technical quality of online–offline integration or all embodied and pedagogical features of physical education. Alternative BLE measurement models are reported in Supplementary Table S3.

Student Learning Engagement (SLE) was measured using the University Student Engagement Inventory (USEI). The USEI was originally developed by Maroco et al. (2016) to assess university students’ engagement as a multidimensional construct. Its Chinese version was validated by She et al. (2023) among 1,504 Chinese university students in an online learning context. The Chinese USEI supported a 14-item, three-factor structure after the removal of one weakly loading reverse-coded item, and the second-order construct showed good reliability and validity. In this study, SLE was treated as a second-order construct comprising three first-order dimensions: Behavioral Engagement (BE, 5 items), Emotional Engagement (EE, 4 items), and Cognitive Engagement (CE, 5 items). Representative items include: for BE, “I usually participate actively in group assignments”; for EE, “I am interested in the work of this course”; and for CE, “When studying, I ask myself questions to make sure I understand the content.”

Civic engagement was measured using the scales developed by Zaff et al. (2017) for college students in mainland China. Civic Actions (CA) consisted of Helping Others (HO; four items), Community Service (CSE; four items), and Acting on Social Problems (ASP; six items). Civic Intentions (CI) consisted of Civic Salience (CSA; four items) and Civic Responsibilities (CIR; four items). Representative items were “I donate money or supplies to people in need” for HO, “I participate in public services organized by NGOs” for CSE, “When I identify a social or community problem, I discuss possible solutions with others” for ASP, “Participating in activities that affect social development is important to me” for CSA, and “I take action to maintain social justice” for CIR. The original four-point response formats were harmonized into a five-point format while retaining item meaning and scoring direction. CA is interpreted as self-reported general civic action, including activities that may occur beyond the focal course. CEng was calculated as (CA + CI)/2.

### 3.3 Analytical strategy

Data analysis was conducted using IBM SPSS Statistics 29.0 and R. The analytic dataset contained 2,035 cases, 69 five-point items, and no missing item responses. Composite scores followed the higher-order structure: BLE = mean(TP, SP, CP), SLE = mean(BE, EE, CE), CA = mean(HO, CSE, ASP), CI = mean(CSA, CIR), and CEng = mean(CA, CI). Internal consistency was assessed using Pearson-based McDonald’s omega total (McDonald, 1999; Dunn et al., 2014); ordinal omega estimates based on polychoric correlations are provided in Supplementary Table S1. Confirmatory factor analysis evaluated 11 correlated first-order factors and the higher-order structure. Composite reliability and average variance extracted were used for convergent validity, and scale-score Fornell–Larcker results were supplemented by higher-order CA–CI diagnostics (Fornell and Larcker, 1981; Hair et al., 2019). Full MLR and WLSMV measurement-model comparisons are reported in Supplementary Table S2, and BLE-specific alternatives in Supplementary Table S3.

Because all constructs were self-reported at one occasion, procedural and statistical method-bias safeguards were used. Participation was voluntary, responses were confidential and analyzed in aggregate, and the questionnaire included clear instructions and an attention check (Podsakoff et al., 2003, 2012). Harman’s (1976) single-factor analysis served only as an initial diagnostic. An MLR one-factor model was compared with the theoretical multi-factor model, followed by an orthogonal unmeasured latent method construct with equal method loadings. A freely estimated method-loading model was evaluated only for admissibility. These analyses assess a general common response factor; they do not directly measure socially desirable responding or demand characteristics specific to the ideological and political education context.

Structural equation modeling was used to estimate the hypothesized associations among BLE, SLE, CA, and CI. The primary 69-item hierarchical model used robust maximum likelihood (MLR), which provides robust standard errors and fit statistics when multivariate normality cannot be assumed. The analysis was repeated with WLSMV after treating all five-point indicators as ordered categorical variables. Model fit was evaluated using robust *χ*^2^, CFI, TLI, SRMR, and RMSEA with its 90% confidence interval (Hu and Bentler, 1999; Kline, 2016). Standardized and unstandardized path estimates, robust standard errors, confidence intervals, and *R*^2^ were reported. A score-level MLR model and path-equality tests provided additional sensitivity evidence, while CA–CI overlap was examined through their latent correlation, broad-item HTMT, and a model constraining the latent correlation to 1. The estimator and model-level comparisons are reported in Supplementary Tables S4, S7.

Indirect associations from BLE to CA and CI through SLE were evaluated in the 69-item MLR model using robust delta-method confidence intervals. Ordered-indicator WLSMV delta intervals and 20,000-draw parameter-covariance Monte Carlo intervals from the score-level MLR model were used as sensitivity analyses (Preacher and Hayes, 2008). An ancillary 2,000-resample percentile bootstrap was also examined for model admissibility; its intervals were not used as the sole inferential basis. Estimates from different model levels were reported with their scales explicitly identified. Supplementary Tables S5, S6 present the interval comparisons and bootstrap diagnostics.

Hierarchical regression analyses provided an additional score-level sensitivity test. CA, CI, and CEng = (CA + CI)/2 were entered separately as dependent variables. Demographic and background variables were entered before BLE and SLE. Standardized coefficients, *R*^2^, adjusted *R*^2^, Δ*R*^2^, and F statistics were reported. These regressions describe adjusted associations and do not alter the latent-variable results.

Exploratory fuzzy-set qualitative comparative analysis was conducted in R using QCA 3.25 to examine configurations of TP, SP, CP, BE, EE, and CE associated separately with high and non-high CA and CI. Direct calibration used the 95th, 50th, and 5th percentiles as full-membership, crossover, and full-nonmembership anchors. The large-sample strict specification used a case-frequency threshold of 31, a consistency threshold of 0.85, and a PRI threshold of 0.75. Necessity was evaluated against a 0.90 consistency criterion (Ragin, 2008; Schneider and Wagemann, 2012). Directional intermediate solutions were summarized by solution consistency, PRI, coverage, and multiplicity.

Configurational stability was assessed across 960 prespecified combinations of scoring, calibration, frequency, consistency, and PRI choices. The solution unions were also compared with a simple direction-aligned rule requiring at least five of the six conditions to be high or low. A frequency-threshold specification of 1 was retained to examine truth-table occupancy and the relation among complex, parsimonious, and intermediate solutions. These analyses follow recommendations for transparent configurational assessment and for clarifying the distinct roles of SEM and fsQCA (Greckhamer et al., 2018; Karuppiah et al., 2024; Akoğul, 2026). Complete exploratory solutions and sensitivity summaries are reported in Supplementary Tables S9–S12.

## 4 Results

### 4.1 Preliminary analyses

#### 4.1.1 Descriptive statistics, reliability, measurement quality, and correlational analysis

Descriptive statistics, internal consistency, and convergent validity are presented in Table 2. Pearson-based McDonald’s omega total coefficients ranged from 0.805 to 0.956 across the first-order dimensions, higher-order composites, and CEng; ordinal omega coefficients ranged from 0.845 to 0.965 (Supplementary Table S1). All estimates exceeded 0.70. Standardized item loadings ranged from 0.654 to 0.872, and higher-order loadings ranged from 0.735 to 0.792. Composite reliability ranged from 0.742 to 0.950 and AVE from 0.508 to 0.632. The 11-correlated-factor and higher-order measurement models both showed very good absolute fit under MLR and WLSMV (Supplementary Table S2). For BLE, a one-factor model fit poorly, whereas the three-correlated-factor model fit well; the second-order model had identical fit because its higher-order level contained three first-order factors and was just identified. An admissible bifactor model supported a general BLE factor while retaining TP, SP, and CP specificity (Supplementary Table S3). These results support the use of the theoretically specified dimensions and a cautious higher-order summary.

**Table 2:** Descriptive statistics, internal consistency reliability, and convergent validity of study variables.

Construct level	Variable/dimension	M	SD	McDonald’s ωt	Std. factor loading	CR	AVE
First-order dimension	TP	3.582	0.938	0.950	0.735–0.843	0.950	0.593
First-order dimension	SP	3.583	0.889	0.907	0.716–0.756	0.907	0.551
First-order dimension	CP	3.634	0.829	0.931	0.676–0.755	0.931	0.528
Second-order construct	BLE	3.600	0.733	0.956	0.735–0.781	0.797	0.567
First-order dimension	BE	3.400	1.013	0.895	0.682–0.872	0.895	0.632
First-order dimension	EE	3.476	0.943	0.858	0.741–0.858	0.859	0.604
First-order dimension	CE	3.533	0.878	0.863	0.666–0.773	0.863	0.559
Second-order construct	SLE	3.470	0.777	0.911	0.735–0.792	0.809	0.586
First-order dimension	HO	3.630	0.929	0.857	0.731–0.824	0.857	0.600
First-order dimension	CSE	3.463	0.987	0.820	0.706–0.763	0.820	0.532
First-order dimension	ASP	3.425	0.979	0.891	0.712–0.806	0.891	0.576
Second-order construct	CA	3.506	0.781	0.902	0.744–0.757	0.795	0.564
First-order dimension	CSA	3.684	0.753	0.805	0.654–0.742	0.805	0.508
First-order dimension	CIR	3.602	0.960	0.840	0.745–0.770	0.840	0.568
Second-order construct	CI	3.643	0.739	0.848	0.766–0.770	0.742	0.590
Composite score	CEng	3.574	0.697	0.925	—	—	—

M, mean; SD, standard deviation; ωt, Pearson-based McDonald’s omega total; CR, composite reliability; AVE, average variance extracted; Std. factor loading, standardized factor-loading range. First-order loading ranges refer to item loadings; higher-order loading ranges refer to first-order dimensions. CEng = (CA + CI)/2. BLE, blended learning experience; TP, teaching presence; SP, social presence; CP, cognitive presence; SLE, student learning engagement; BE, behavioral engagement; EE, emotional engagement; CE, cognitive engagement; HO, helping others; CSE, community service; ASP, acting on social problems; CSA, civic salience; CIR, civic responsibilities; CA, civic actions; CI, civic intentions; CEng, overall civic engagement.

Table 3 reports scale-score correlations and the Fornell–Larcker assessment. BLE, SLE, CA, and CI were positively correlated, and each diagonal square root of AVE exceeded the corresponding scale-score correlations. The higher-order latent results nevertheless indicated substantial overlap between CA and CI: their latent correlation was approximately 0.992 under both MLR and WLSMV, and fixing this correlation to 1 did not significantly worsen model fit. Broad-item HTMT was 0.783, 95% CI [0.759, 0.804]. CA and CI were therefore retained as theoretically specified scores, but the data do not support strong claims of empirical separation or comparisons of path magnitude between the two outcomes. Full diagnostics and path-equality tests are reported in Supplementary Table S7.

**Table 3:** Scale-score correlations and Fornell–Larcker results for the SEM constructs.

Variable	BLE	SLE	CA	CI
BLE	**0.753**			
SLE	0.397^***^	**0.766**		
CA	0.515^***^	0.473^***^	**0.751**	
CI	0.463^***^	0.444^***^	0.686^***^	**0.768**

Values on the diagonal in bold are the square roots of AVE; off-diagonal values are scale-score correlations. The Fornell–Larcker comparisons apply to these composite scores. Higher-order latent diagnostics indicated substantial CA–CI overlap and are reported in Supplementary Table S7. BLE, blended learning experience; SLE, student learning engagement; CA, civic actions; CI, civic intentions. * *p* < 0.05, ** *p* < 0.01, *** *p* < 0.001.

#### 4.1.2 Measurement models and common method sensitivity

All constructs were measured by self-report at one occasion, making a dominant common response factor and context-specific socially desirable responding relevant interpretive concerns. Procedural safeguards and several statistical diagnostics were therefore considered together.

Participants were informed that the survey was for academic research, participation was voluntary, responses would be analyzed in aggregate, and participation would not affect grades or instructor evaluations. The online format, established instruments, clear instructions, and attention check were intended to reduce evaluation apprehension and inattentive responding. Recruitment through course channels and the value-laden content of ideological and political education could nevertheless encourage acquiescent or socially desirable responses, which cannot be directly quantified without a dedicated measure.

Harman’s unrotated exploratory analysis extracted 11 factors with eigenvalues above 1. They explained 63.762% of the variance, and the first factor accounted for 28.490%. This initial diagnostic did not indicate that one factor dominated the item covariance, but it was not treated as sufficient evidence against common method bias.

The MLR one-factor model fit poorly, whereas the 11-correlated-factor and higher-order models fit well (Table 4). The one-factor model yielded robust CFI = 0.502, robust TLI = 0.487, SRMR = 0.106, and robust RMSEA = 0.092; the 11-factor model yielded robust CFI = 0.992, robust TLI = 0.991, SRMR = 0.017, and robust RMSEA = 0.012. In the admissible equal-loading orthogonal method-factor model, estimated method variance was effectively zero and the five structural paths were unchanged to three decimals. A freely estimated method-loading model produced a negative item residual variance and was not interpreted. The combined evidence does not indicate a dominant general method factor, while leaving context-specific social desirability and demand characteristics unresolved (Supplementary Table S8).

**Table 4:** Fit of alternative measurement models.

Model	Structure	Robust *χ*^2^ (df)	Robust CFI	Robust TLI	SRMR	Robust RMSEA	90% CI
M0	One factor	39561.362 (2277)	0.502	0.487	0.106	0.092	[0.091, 0.093]
M1	11 correlated first-order factors	2839.290 (2222)	0.992	0.991	0.017	0.012	[0.011, 0.013]
M2	Four higher-order constructs	2937.479 (2261)	0.991	0.991	0.023	0.012	[0.011, 0.014]

Robust maximum likelihood estimation was used. M1 specifies TP, SP, CP, BE, EE, CE, HO, CSE, ASP, CSA, and CIR as correlated first-order factors. M2 specifies the four higher-order constructs BLE, SLE, CA, and CI. WLSMV results and nested comparisons are reported in Supplementary Table S2. CFI, comparative fit index; TLI, Tucker–Lewis index; SRMR, standardized root mean square residual; RMSEA, root mean square error of approximation.

### 4.2 Structural equation modeling results

#### 4.2.1 Model fit and path analysis

The 69-item hierarchical structural model was estimated with MLR. It showed good fit: robust χ^2^(2261) = 2937.479, robust CFI = 0.991, robust TLI = 0.991, SRMR = 0.023, and robust RMSEA = 0.012, 90% CI [0.011, 0.014].

The structural estimates are presented in Table 5 and Figure 2. BLE was positively associated with CA (B = 0.752, robust SE = 0.058, *β* = 0.490, *p* < 0.001), CI (B = 0.663, robust SE = 0.062, *β* = 0.447, *p* < 0.001), and SLE (B = 0.625, robust SE = 0.039, *β* = 0.530, *p* < 0.001), supporting H1a, H1b, and H2. SLE was positively associated with CA (B = 0.488, robust SE = 0.046, *β* = 0.375, *p* < 0.001) and CI (B = 0.498, robust SE = 0.050, *β* = 0.396, *p* < 0.001), supporting H3a and H3b.

**Table 5:** Structural path estimates under robust maximum likelihood.

Path	Hypothesis	B	Robust SE	95% CI	*β*	*p*
BLE → CA	H1a	0.752	0.058	[0.637, 0.866]	0.490	< 0.001
BLE → CI	H1b	0.663	0.062	[0.542, 0.785]	0.447	< 0.001
BLE → SLE	H2	0.625	0.039	[0.549, 0.702]	0.530	< 0.001
SLE → CA	H3a	0.488	0.046	[0.398, 0.577]	0.375	< 0.001
SLE → CI	H3b	0.498	0.050	[0.401, 0.596]	0.396	< 0.001

B, unstandardized coefficient; SE, robust standard error; CI, confidence interval; *β*, standardized coefficient. BLE, blended learning experience; SLE, student learning engagement; CA, civic actions; CI, civic intentions.

**Figure 2:**
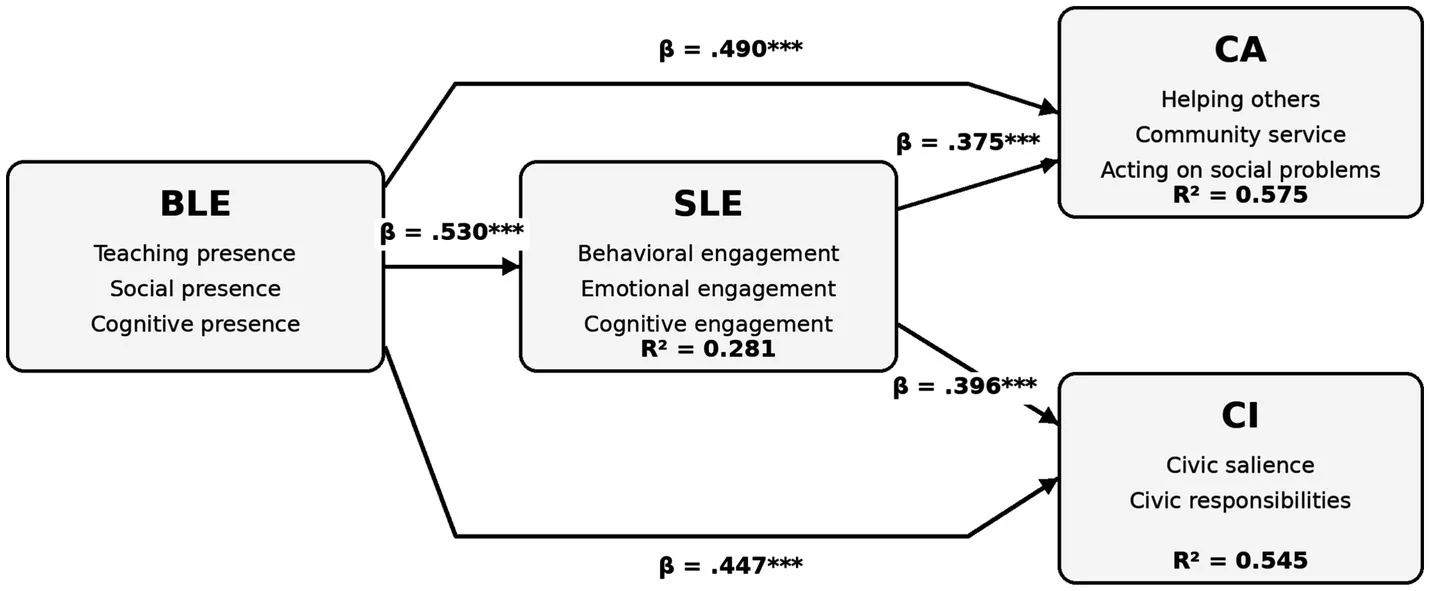
Standardized structural estimates under robust maximum likelihood. Values beside arrows are standardized coefficients; values inside endogenous constructs are *R*^2^. The residual covariance between CA and CI was estimated but is not shown. BLE, blended learning experience; SLE, student learning engagement; CA, civic actions; CI, civic intentions. *** *p* < 0.001.

BLE accounted for 28.1% of the variance in SLE. BLE and SLE jointly accounted for 57.5% of the variance in CA and 54.5% of the variance in CI. All five hypothesized structural associations were positive and statistically significant.

#### 4.2.2 Robust indirect-association analysis

Indirect associations were evaluated with robust delta-method intervals in the 69-item MLR model. Statistical support was defined by a 95% confidence interval that excluded zero. Ordered-indicator WLSMV delta intervals and score-level Monte Carlo intervals served as sensitivity analyses.

As shown in Table 6, BLE had a positive indirect association with CA through SLE, B = 0.305, robust SE = 0.031, 95% CI [0.244, 0.365], standardized *β* = 0.199, supporting H4a. The corresponding indirect association with CI was also positive, B = 0.312, robust SE = 0.034, 95% CI [0.245, 0.378], standardized *β* = 0.210, supporting H4b. WLSMV delta intervals and 20,000-draw Monte Carlo intervals led to the same conclusion (Supplementary Table S5). In an ancillary score-level percentile bootstrap, all 2,000 resamples returned numerical estimates, but 737 (36.85%) were nonadmissible because the latent residual covariance matrix was non-positive definite and the absolute CA–CI residual correlation exceeded 1. Accordingly, admissible-only bootstrap intervals were treated as sensitivity evidence rather than the primary inferential basis (Supplementary Table S6).

**Table 6:** Indirect associations through student learning engagement.

Path	Hypothesis	B	Robust SE	95% CI	*β*	*p*
BLE → SLE → CA	H4a	0.305	0.031	[0.244, 0.365]	0.199	< 0.001
BLE → SLE → CI	H4b	0.312	0.034	[0.245, 0.378]	0.210	< 0.001

B, unstandardized indirect association; SE, robust standard error; CI, confidence interval; *β*, standardized indirect association. Estimates are from the 69-item MLR model. BLE, blended learning experience; SLE, student learning engagement; CA, civic actions; CI, civic intentions.

#### 4.2.3 Robustness analyses

Estimator and model-level sensitivity analyses supported the primary results. The score-level MLR coefficients were 0.527, 0.492, 0.450, 0.372, and 0.394, and the ordered-indicator WLSMV coefficients were 0.538, 0.476, 0.432, 0.379, and 0.405 for BLE → SLE, BLE → CA, BLE → CI, SLE → CA, and SLE → CI, respectively; all *p* values were below 0.001. The largest difference from the item-level MLR estimates was 0.015, and *R*^2^ conclusions were comparable (Supplementary Table S4). Hierarchical regressions supplied an additional adjusted score-level analysis, with CA, CI, and CEng entered as dependent variables.

As shown in Table 7, adding BLE and SLE increased explained variance by 0.243 for CA, 0.199 for CI, and 0.262 for CEng. In the full models, BLE and SLE were positively associated with all three civic scores after adjustment for the reported demographic and background variables. These score-level results were consistent with the direction of the latent-variable estimates.

**Table 7:** Adjusted associations with civic engagement scores: hierarchical regression sensitivity analysis.

Variable	Model 1: CA	Model 2: CA	Model 3: CI	Model 4: CI	Model 5: CEng	Model 6: CEng
Gender	0.100^***^	0.049^**^	0.113^***^	0.067^***^	0.116^***^	0.063^***^
Age	0.192^***^	0.085^***^	0.196^***^	0.099^***^	0.211^***^	0.100^***^
Year of study	0.009	0.016	−0.004	0.002	0.003	0.010
Academic discipline	0.031	0.011	0.035	0.018	0.036	0.016
Place of origin	−0.063^**^	−0.063^***^	−0.044^*^	−0.044^*^	−0.059^**^	−0.058^***^
Only-child status	0.176^***^	0.090^***^	0.129^***^	0.051^**^	0.167^***^	0.078^***^
Ethnicity	0.188^***^	0.096^***^	0.194^***^	0.111^***^	0.208^***^	0.113^***^
Weekly extracurricular physical activity frequency	−0.013	−0.019	−0.014	−0.020	−0.015	−0.021
Prior blended learning experience	0.017	−0.003	0.005	−0.014	0.012	−0.009
BLE		0.351^***^		0.301^***^		0.356^***^
SLE		0.280^***^		0.271^***^		0.300^***^
*R* ^2^	0.141	0.384	0.129	0.328	0.159	0.421
Adjusted *R*^2^	0.137	0.380	0.125	0.324	0.155	0.418
Δ*R*^2^	0.141	0.243	0.129	0.199	0.159	0.262
*F*	36.826^***^	114.524^***^	33.267^***^	89.663^***^	42.580^***^	133.887^***^

Standardized regression coefficients are reported. Models 1, 3, and 5 include only control variables. Models 2, 4, and 6 add BLE and SLE. CEng = (CA + CI)/2. CA, civic actions; CI, civic intentions; CEng, overall civic engagement; BLE, blended learning experience; SLE, student learning engagement. * *p* < 0.05, ** *p* < 0.01, *** *p* < 0.001.

### 4.3 Exploratory configurational analysis

No individual condition or its absence met the 0.90 necessity threshold for high or non-high CA or CI. Under the large-sample strict specification, the maximum necessity consistency was 0.756; across all scoring and calibration sensitivity analyses, the maximum was 0.827.

The strict directional intermediate solutions were dominated by conjunctions of several simultaneously high or low conditions. High CA was associated with TP*SP*BE*EE*CE, and high CI with TP*CP*BE*EE*CE or TP*SP*BE*EE*CE. The corresponding non-high solutions were also composed mainly of several absent conditions. Solution consistency ranged from 0.875 to 0.919 and solution coverage from 0.396 to 0.532. Detailed expressions, PRI values, and multiplicity information are reported in Supplementary Table S9.

The solutions were sensitive to prespecified analytical choices. Across 960 specifications, successful runs yielded 37 unique expressions for CA, 23 for non-high CA, 19 logic-normalized expressions for CI, and 7 for non-high CI; some CA and CI specifications produced computational failures or no positive truth-table rows (Supplementary Table S10). Strict solution unions correlated 0.741–0.801 with a simple direction-aligned rule requiring at least five high or low conditions, with Jaccard overlap of 0.520–0.715 (Supplementary Table S11). Under the frequency threshold of 1, all 64 logical rows were represented, leaving no logical remainders; complex, parsimonious, and intermediate solutions therefore coincided. The corresponding frequency-threshold-1 solutions are shown in Supplementary Table S12. The configurational findings are presented as exploratory descriptions of broadly favorable or unfavorable profiles.

## 5 Discussion

### 5.1 Interpretation of the SEM findings

The primary SEM examined associations among CoI-based perceived blended learning experience, SLE, CA, and CI in university physical education incorporating ideological and political education. BLE was positively associated with SLE and both civic scores, and SLE was positively associated with CA and CI. Positive indirect associations through SLE were also supported across MLR, ordered-indicator WLSMV, and Monte Carlo interval estimation. The close agreement among estimators indicates that the substantive pattern was not dependent on treating five-point indicators as continuous under conventional maximum likelihood.

The positive BLE–SLE association is consistent with the Community of Inquiry framework, which organizes perceived educational experience around teaching, social, and cognitive presence (Garrison et al., 2000; Arbaugh et al., 2008). It also accords with evidence that blended learning in higher education is most relevant when instructional organization, interaction, and reflective activity are coherently arranged (Means et al., 2013; Bernard et al., 2014). In Chinese university physical education, Yu et al. (2025) likewise reported a positive relationship between blended learning and learning engagement. The present results extend that comparison to civic outcome scores while retaining a narrower measurement interpretation: the CoI scale captured students’ perceived presence in the focal course, not an objective audit of online–offline integration or all PE-specific design features.

Associations with CA and CI are also compatible with research linking physical education integrated with civic or responsibility-oriented content to prosocial and social-responsibility outcomes (Huang et al., 2023; Ma et al., 2026). The present civic-action items, however, include helping, NGO service, and responses to social problems that may occur outside the course. Their association with course experience can reflect broader prosocial orientation, pre-existing civic involvement, access to service opportunities, and a general tendency to evaluate educational and civic domains favorably. This ecological distance makes the CA findings relevant but necessarily cautious.

CA and CI were retained as theoretically specified scores, yet their higher-order latent correlation approached unity and constraining it to 1 did not worsen model fit. Robust equality tests also detected no difference between the BLE paths to CA and CI or between the SLE paths to the two outcomes (Supplementary Table S7). The findings therefore support a broad civic-engagement association more clearly than distinct outcome-specific patterns. Separate reporting remains useful for continuity with the validated instrument and for displaying the behavioral and intentional content of the scales, but relative path magnitudes should not be interpreted.

### 5.2 Indirect associations through student learning engagement

SLE showed positive indirect associations linking BLE with both civic scores in the specified model. The direct BLE associations and the indirect components through SLE were statistically different from zero, and the indirect conclusions were stable under robust delta and Monte Carlo intervals. This pattern indicates that behavioral, emotional, and cognitive engagement contribute to the statistical association between perceived learning presence and civic outcomes; it does not demonstrate a temporally ordered mediating process.

The result is consistent with the multidimensional view of student engagement (Fredricks et al., 2004) and with higher education accounts that position engagement at the interface between students and their learning environments (Kahu, 2013). Behavioral participation, affective connection, and cognitive investment are plausible reasons for covariation between favorable course perceptions and civic orientations (Li, 2025). Related physical education research has also linked supportive blended environments with engagement and responsibility-related outcomes (Li and Zeng, 2025; Ma et al., 2026).

The cross-sectional design leaves several directions compatible with the observed covariance. Favorable perceived learning presence may coincide with stronger engagement; students who are generally more engaged may evaluate teaching and peer interaction more positively; and students with stronger pre-existing prosocial or civic orientations may report both greater course engagement and higher civic scores. Longitudinal measurement before, during, and after the course is required to distinguish these possibilities and to test whether changes in engagement precede later civic outcomes.

The SLE associations remain educationally meaningful in physical education because the course combines bodily practice, cooperation, rules, collective responsibility, and opportunities for reflective discussion. Reviews have linked well-designed physical education and school sport with social, affective, and cognitive development (Bailey et al., 2009). The present results add evidence that students who report stronger teaching, social, and cognitive presence also tend to report stronger learning engagement and civic orientations within a university context integrating ideological and political education.

### 5.3 Exploratory configurational findings

The exploratory configurational analysis aligned the first-order conditions with CA and CI rather than using a single unmatched civic composite. No condition met the conventional necessity threshold. Under the strict specification, high outcomes were associated mainly with conjunctions containing five of the six favorable conditions, while non-high outcomes were associated mainly with several jointly absent conditions.

This pattern offers limited evidence for strong substitution or highly differentiated equifinal routes. The strict CA solution required TP, SP, BE, EE, and CE, and both strict CI solution terms required five favorable conditions. Unique expressions varied substantially across the 960 sensitivity specifications, and the configuration unions overlapped moderately to strongly with a simple rule representing many conditions jointly high or low.

The frequency-threshold analysis also clarified why the complex, parsimonious, and intermediate solutions coincided under the threshold-1 specification: all 64 logical combinations were represented, leaving no logical remainders for counterfactual simplification. The identical solution types were therefore a consequence of truth-table occupancy and did not indicate that every reported condition was uniquely robust.

The configurational findings are best treated as exploratory context for the SEM. They indicate that broadly favorable profiles across learning presence and engagement tend to accompany higher civic scores, but they add limited stable information beyond that general gradient. This restrained interpretation is consistent with integrated-method applications in which the contribution of fsQCA depends on transparent outcome alignment, truth-table reporting, and sensitivity analysis (Karuppiah et al., 2024; Akoğul, 2026).

### 5.4 Theoretical implications

The findings have theoretical implications within the boundaries of the sample, design, and measurement approach. The CoI framework provided a coherent vocabulary for teaching, social, and cognitive presence in university physical education incorporating ideological and political education. The alternative measurement models rejected a single undifferentiated BLE factor and supported retaining TP, SP, and CP, while the bifactor evidence also supported a cautious higher-order summary. CoI remains a representation of perceived learning presence and does not exhaust the bodily, skill-based, technological, and pedagogical features of physical education.

The model also places multidimensional engagement within the association between perceived learning presence and civic outcome scores. Behavioral, emotional, and cognitive engagement were represented as related dimensions rather than isolated indicators. Their stable associations across MLR and WLSMV support engagement as a useful organizing construct for research on blended university physical education, while the design does not establish it as a causal mechanism.

The separate CA and CI scores preserve the behavioral and intentional domains of the civic engagement instrument developed for mainland Chinese college students (Zaff et al., 2017). Their near-unity higher-order correlation in the present sample, however, indicates that the two domains were not strongly distinguishable at the latent level. Theoretical distinctions between action and intention should therefore be accompanied by explicit empirical tests rather than inferred from subscale labels alone.

Methodologically, the study illustrates the value of combining robust latent-variable estimation with transparent sensitivity analysis. MLR, ordered-indicator WLSMV, Monte Carlo intervals, and an admissible method-factor specification converged on the same structural pattern. The fsQCA results also show why configurational findings require aligned outcomes, adequate frequency thresholds, disclosure of truth-table occupancy, and comparison across analytical choices before claims of equifinality or substitution are made.

### 5.5 Practical implications

The practical implications are design-oriented considerations rather than tested intervention effects. Blended university physical education may benefit from coherent connections among classroom instruction, online resources, physical practice, extracurricular participation, and reflective tasks (Garrison and Kanuka, 2004; Garrison and Vaughan, 2008; Vaughan et al., 2023). The results are consistent with attention to instructional organization, interaction, and reflection rather than technology use alone.

Teaching presence can be reflected in clear goals, task organization, feedback, and guidance; social presence in peer interaction, team-based activity, and belonging; and cognitive presence in reflective questions, problem-based tasks, and connections between sport experience and broader social meanings (Caskurlu, 2018). The three-dimensional measurement results favor coordinated attention to these domains over treating perceived course quality as a single undifferentiated attribute.

Behavioral, emotional, and cognitive engagement can also be monitored as course-process indicators. Participation in tasks, connection with the course community, and reflection on cooperation, fairness, rules, and responsibility offer concrete targets for formative course evaluation. Their observed associations do not show that raising one engagement dimension will independently produce civic outcomes, but they identify domains that can be considered alongside learning presence.

The content of CA and CI also has practical relevance. Intentional and value-oriented responses can be discussed within coursework, whereas civic action depends on genuine opportunities, organizational access, time, and voluntary participation (Doolittle and Faul, 2013). Links with campus sport services, peer-support activities, community fitness programs, volunteer events, or student-led health promotion can provide concrete settings for participation (Whitley and Yoder, 2015). Such opportunities should remain voluntary and should not be treated as evidence that attitudes will automatically become behavior.

The exploratory configurational results favor a multicomponent view but do not support selecting isolated “alternative pathways” from the current data. Higher civic scores generally coincided with many favorable learning-presence and engagement conditions, and the solutions changed across analytical specifications. Course development should therefore be guided by pedagogical coherence and local feasibility rather than by treating any single fsQCA expression as a validated instructional recipe.

### 5.6 Limitations and future directions

Several limitations should be noted. The cross-sectional survey cannot establish temporal ordering among BLE, SLE, CA, and CI. Direct and indirect arrows represent theory-guided statistical associations, and reciprocal or common-cause explanations remain possible (Maxwell and Cole, 2007). Longitudinal designs with pre-course, during-course, and post-course measurement are needed to examine whether changes in perceived learning experience and engagement precede later civic outcomes.

All constructs were self-reported at one occasion. The equal-loading ULMC did not change the structural estimates and did not indicate a dominant general method factor, but a freely estimated method model was inadmissible. More importantly, recruitment through instructors and course channels, together with questions about civic responsibility in an ideological and political education context, may encourage acquiescent or socially desirable responding. No dedicated social-desirability scale or prespecified marker was available, so this context-specific bias cannot be excluded. Future studies should combine surveys with instructor or peer ratings, learning-platform records, participation logs, documented civic involvement, and a direct social-desirability measure.

The sample comprised undergraduate students from 15 universities in Sichuan Province. The multi-site design introduced regional institutional variation, but the findings may not generalize to other provinces, institutional types, or curricular systems. Future research should compare regions, university types, and models of physical education curriculum implementation.

The study focused on university physical education courses incorporating ideological and political education. Physical education has distinctive bodily, cooperative, competitive, and extracurricular features, so the results should not be generalized directly to other courses. The CoI instrument also measured perceived teaching, social, and cognitive presence rather than objective online–offline integration, sport-specific pedagogy, or course-level implementation. Course observations and instructor-level design data would strengthen construct and contextual validity.

The civic engagement scale used harmonized five-point response options rather than the original response formats, which may affect comparability with prior studies (He et al., 2017; Zhang and Savalei, 2024). CA included self-reported general civic activities that are distal from a single physical education course and may reflect prior prosocial disposition or opportunity structures. CA and CI also showed near-unity higher-order overlap in this sample. Further validation should compare response formats, incorporate behavioral records and baseline civic involvement, and test whether action and intention are distinguishable across samples and time.

The exploratory fsQCA results depended substantially on scoring, calibration, and truth-table thresholds. Many solution terms contained five jointly high or low conditions, unique expressions varied across the 960 specifications, and configuration unions overlapped with a simple majority rule. The findings warrant an exploratory interpretation. Independent replication with theoretically anchored calibration and complete truth-table reporting is needed before stronger configurational implications are drawn.

Students were nested within universities and courses, but no university, class, or instructor identifier was available in the analytic file for estimating intraclass correlations or cluster-robust models. Shared instructional environments may therefore contribute to the observed associations. Future data collection should preserve de-identified course and instructor identifiers and combine student-level perceptions with course-level design information.

## 6 Conclusion

This cross-sectional multi-site study found consistent positive associations among CoI-based perceived blended learning experience, student learning engagement, and self-reported civic action and intention scores in university physical education incorporating ideological and political education. Robust MLR and ordered-indicator WLSMV supported all five structural associations, and robust delta and Monte Carlo intervals supported positive indirect associations through student learning engagement. CA and CI showed substantial higher-order overlap, and the exploratory fsQCA solutions were sensitive to analytical choices and largely reflected profiles with many conditions jointly high or low. The most defensible interpretation is therefore an associational one: coordinated teaching, social, cognitive, and engagement experiences were related to civic orientations in this sample, while temporal sequence, causality, course-specific effects on distal civic behavior, and context-specific social desirability remain to be established through longitudinal, multi-source, and course-level research.

## Data Availability

The original contributions presented in the study are included in the article/Supplementary material, further inquiries can be directed to the corresponding author.
