# Supplementary material for: Blended learning experience, student engagement, and civic engagement in university physical education with ideological and political education
Source: Front Psychol. 2026 Sep 25;17:1888349. doi: 10.3389/fpsyg.2026.1888349 (PMC13649470; doi:10.3389/fpsyg.2026.1888349)
Supplement: Supplementary file 1 [file Table_1.DOCX]

**Supplementary materials**

*Blended learning experience, student engagement, and civic engagement in university physical education with ideological and political education*

**Table S1. Composite scoring and internal consistency estimates**

| **Construct** | **Score calculation** | **Items** | **McDonald’s ωt** | **Ordinal ω** |
| --- | --- | --- | --- | --- |
| TP | Mean of 13 TP items | 13 | .950 | .960 |
| SP | Mean of 8 SP items | 8 | .907 | .931 |
| CP | Mean of 12 CP items | 12 | .931 | .950 |
| BLE | Mean of TP, SP, and CP | 33 | .956 | .965 |
| BE | Mean of 5 BE items | 5 | .895 | .914 |
| EE | Mean of 4 EE items | 4 | .858 | .893 |
| CE | Mean of 5 CE items | 5 | .863 | .896 |
| SLE | Mean of BE, EE, and CE | 14 | .911 | .925 |
| HO | Mean of 4 HO items | 4 | .857 | .898 |
| CSE | Mean of 4 CSE items | 4 | .820 | .861 |
| ASP | Mean of 6 ASP items | 6 | .891 | .909 |
| CA | Mean of HO, CSE, and ASP | 14 | .902 | .918 |
| CSA | Mean of 4 CSA items | 4 | .805 | .845 |
| CIR | Mean of 4 CIR items | 4 | .840 | .870 |
| CI | Mean of CSA and CIR | 8 | .848 | .873 |
| CEng | Mean of CA and CI | 22 | .925 | .937 |

Note. ωt is Pearson-based McDonald’s omega total; ordinal omega is based on polychoric item correlations. BLE = Blended Learning Experience; SLE = Student Learning Engagement; CA = Civic Actions; CI = Civic Intentions; CEng = overall Civic Engagement. CEng = (CA + CI) / 2.

**Table S2. Fit of full measurement models**

| **Model** | **Structure** | **Estimator** | **Robust χ²** | **df** | **CFI** | **TLI** | **RMSEA [90% CI]** | **SRMR** |
| --- | --- | --- | --- | --- | --- | --- | --- | --- |
| M0 | One factor | MLR | 39561.362 | 2277 | .502 | .487 | .092 [.091, .093] | .106 |
| M0 | One factor | WLSMV | 57904.433 | 2277 | .475 | .459 | .106 [.105, .107] | .122 |
| M1 | 11 correlated first-order factors | MLR | 2839.290 | 2222 | .992 | .991 | .012 [.011, .013] | .017 |
| M1 | 11 correlated first-order factors | WLSMV | 2701.354 | 2222 | .989 | .988 | .016 [.014, .017] | .017 |
| M2 | Four higher-order constructs | MLR | 2937.479 | 2261 | .991 | .991 | .012 [.011, .014] | .023 |
| M2 | Four higher-order constructs | WLSMV | 3140.273 | 2261 | .988 | .988 | .016 [.014, .018] | .023 |
| M3 | Structural model | MLR | 2937.479 | 2261 | .991 | .991 | .012 [.011, .014] | .023 |
| M3 | Structural model | WLSMV | 3140.273 | 2261 | .988 | .988 | .016 [.014, .018] | .023 |

Note. M1 contains TP, SP, CP, BE, EE, CE, HO, CSE, ASP, CSA, and CIR as correlated first-order factors. M2 contains BLE, SLE, CA, and CI as higher-order constructs. The M2-versus-M1 restrictions reduced fit under MLR, Δχ²(39) = 97.343, p < .001, and WLSMV, Δχ²(39) = 145.928, p < .001, while absolute fit remained strong. M2 and M3 are observationally equivalent reparameterizations.

**Table S3. Fit and bifactor diagnostics for alternative BLE measurement models**

| **Model** | **Structure** | **Estimator** | **Robust χ²** | **df** | **CFI** | **TLI** | **RMSEA [90% CI]** | **SRMR** | **ωH** | **ECV** | **PUC** | **Determinacy** |
| --- | --- | --- | --- | --- | --- | --- | --- | --- | --- | --- | --- | --- |
| B1 | Single BLE factor | MLR | 11988.117 | 495 | .694 | .673 | .112 [.110, .113] | .109 | — | — | — | — |
| B1 | Single BLE factor | WLSMV | 16743.350 | 495 | .661 | .639 | .132 [.130, .135] | .125 | — | — | — | — |
| B2 | Correlated TP, SP, and CP factors | MLR | 965.704 | 492 | .987 | .987 | .023 [.021, .025] | .018 | — | — | — | — |
| B2 | Correlated TP, SP, and CP factors | WLSMV | 870.030 | 492 | .983 | .982 | .030 [.027, .032] | .018 | — | — | — | — |
| B3 | Second-order BLE factor | MLR | 965.704 | 492 | .987 | .987 | .023 [.021, .025] | .018 | — | — | — | — |
| B3 | Second-order BLE factor | WLSMV | 870.030 | 492 | .983 | .982 | .030 [.027, .032] | .018 | — | — | — | — |
| B4 | General BLE factor plus TP, SP, and CP specific factors | MLR | 910.806 | 462 | .988 | .986 | .023 [.021, .025] | .014 | .770 | .572 | .674 | .882 |
| B4 | General BLE factor plus TP, SP, and CP specific factors | WLSMV | 920.316 | 462 | .984 | .982 | .030 [.027, .033] | .015 | .782 | .581 | .674 | .890 |

Note. B2 and B3 have identical fit because a second-order factor with three first-order indicators is just identified. B4 was admissible under both estimators. ωH = omega hierarchical; ECV = explained common variance; PUC = percentage of uncontaminated correlations.

**Table S4. Structural estimates across analysis levels and estimators**

| **Analysis level** | **Estimator** | **Metric** | **Estimate** | **p** |
| --- | --- | --- | --- | --- |
| 11-dimension score model | MLR | BLE → SLE | 0.527 | < .001 |
| 11-dimension score model | MLR | BLE → CA | 0.492 | < .001 |
| 11-dimension score model | MLR | BLE → CI | 0.450 | < .001 |
| 11-dimension score model | MLR | SLE → CA | 0.372 | < .001 |
| 11-dimension score model | MLR | SLE → CI | 0.394 | < .001 |
| 11-dimension score model | MLR | R²: SLE | 0.277 | — |
| 11-dimension score model | MLR | R²: CA | 0.573 | — |
| 11-dimension score model | MLR | R²: CI | 0.544 | — |
| 69-item hierarchical model | MLR | BLE → SLE | 0.530 | < .001 |
| 69-item hierarchical model | MLR | BLE → CA | 0.490 | < .001 |
| 69-item hierarchical model | MLR | BLE → CI | 0.447 | < .001 |
| 69-item hierarchical model | MLR | SLE → CA | 0.375 | < .001 |
| 69-item hierarchical model | MLR | SLE → CI | 0.396 | < .001 |
| 69-item hierarchical model | MLR | R²: SLE | 0.281 | — |
| 69-item hierarchical model | MLR | R²: CA | 0.575 | — |
| 69-item hierarchical model | MLR | R²: CI | 0.545 | — |
| 69-item hierarchical model | WLSMV | BLE → SLE | 0.538 | < .001 |
| 69-item hierarchical model | WLSMV | BLE → CA | 0.476 | < .001 |
| 69-item hierarchical model | WLSMV | BLE → CI | 0.432 | < .001 |
| 69-item hierarchical model | WLSMV | SLE → CA | 0.379 | < .001 |
| 69-item hierarchical model | WLSMV | SLE → CI | 0.405 | < .001 |
| 69-item hierarchical model | WLSMV | R²: SLE | 0.290 | — |
| 69-item hierarchical model | WLSMV | R²: CA | 0.565 | — |
| 69-item hierarchical model | WLSMV | R²: CI | 0.540 | — |

Note. Path entries are standardized coefficients. R² entries are explained-variance estimates. All five structural paths retained the same direction and significance conclusion. The maximum difference between the item-level MLR and WLSMV standardized paths was .015.

**Table S5. Indirect associations across robust interval methods**

| **Model level** | **Estimator** | **Interval method** | **Path** | **Scale** | **Estimate** | **SE** | **95% CI** | **Std. β** | **Valid draws** |
| --- | --- | --- | --- | --- | --- | --- | --- | --- | --- |
| 69-item hierarchical model | MLR | Robust delta | BLE → SLE → CA | Unstandardized B | .305 | .031 | [.244, .365] | .199 | — |
| 69-item hierarchical model | MLR | Robust delta | BLE → SLE → CI | Unstandardized B | .312 | .034 | [.245, .378] | .210 | — |
| 69-item hierarchical model | WLSMV | Robust delta | BLE → SLE → CA | Unstandardized B | .310 | .031 | [.248, .371] | .204 | — |
| 69-item hierarchical model | WLSMV | Robust delta | BLE → SLE → CI | Unstandardized B | .322 | .034 | [.254, .389] | .218 | — |
| 11-dimension score model | MLR | 20,000-draw Monte Carlo | BLE → SLE → CA | Standardized β | .196 | .018 | [.162, .231] | .196 | 20,000 |
| 11-dimension score model | MLR | 20,000-draw Monte Carlo | BLE → SLE → CI | Standardized β | .207 | .020 | [.169, .246] | .207 | 20,000 |
| 11-dimension score model | MLR | Percentile bootstrap; admissible only | BLE → SLE → CA | Standardized β | .196 | .018 | [.161, .233] | .196 | 1,263 |
| 11-dimension score model | MLR | Percentile bootstrap; admissible only | BLE → SLE → CI | Standardized β | .207 | .020 | [.168, .248] | .207 | 1,263 |

Note. Item-level delta-method estimates are unstandardized B with standardized β reported separately. Score-level Monte Carlo and bootstrap estimates are standardized. Estimates from different model levels and scales are not directly interchangeable.

**Table S6. Bootstrap admissibility diagnostics**

| **Diagnostic** | **Count** | **Percentage of requested resamples** |
| --- | --- | --- |
| Requested resamples | 2,000 | 100.00 |
| Failed fits | 0 | 0.00 |
| Complete numerical effect rows | 2,000 | 100.00 |
| Nonadmissible solutions | 737 | 36.85 |
| Admissible solutions | 1,263 | 63.15 |
| Negative observed residual variance | 0 | 0.00 |
| Negative latent variance | 0 | 0.00 |
| Latent residual covariance matrix not positive definite | 737 | 36.85 |
| \|CA–CI residual correlation\| > 1 | 737 | 36.85 |
| Latent covariance matrix not positive definite | 737 | 36.85 |
| Absolute latent correlation > 1 | 671 | 33.55 |

Note. All 2,000 resamples returned finite numerical indirect estimates, but 737 solutions were nonadmissible. Percentile intervals based on the 1,263 admissible draws are therefore reported only as sensitivity evidence.

**Table S7. CA–CI overlap and path-equality diagnostics**

| **Analysis** | **Estimator** | **Statistic** | **df** | **Value** | **95% CI** | **p** | **Interpretation** |
| --- | --- | --- | --- | --- | --- | --- | --- |
| CA–CI latent correlation | MLR | Estimate | — | .992 | — | — | Near-unity correlation |
| CA–CI latent correlation | WLSMV | Estimate | — | .992 | — | — | Near-unity correlation |
| CA–CI broad-item HTMT | Pearson; 1,000 bootstrap draws | Estimate | — | .783 | [.759, .804] | — | Substantial overlap |
| Latent correlation fixed to 1 versus freely estimated | MLR | Δχ² | 1 | .202 | — | .653 | No detectable loss of fit |
| Latent correlation fixed to 1 versus freely estimated | WLSMV | Δχ² | 1 | .192 | — | .661 | No detectable loss of fit |
| BLE paths to CA and CI constrained equal | MLR | Δχ² | 1 | 1.952 | — | .162 | No detectable difference |
| SLE paths to CA and CI constrained equal | MLR | Δχ² | 1 | .061 | — | .805 | No detectable difference |
| Both outcome-path pairs constrained equal | MLR | Δχ² | 2 | 1.983 | — | .371 | No detectable joint difference |
| BLE paths to CA and CI constrained equal | WLSMV | Δχ² | 1 | 1.656 | — | .198 | No detectable difference |
| SLE paths to CA and CI constrained equal | WLSMV | Δχ² | 1 | .066 | — | .797 | No detectable difference |
| Both outcome-path pairs constrained equal | WLSMV | Δχ² | 2 | 1.682 | — | .431 | No detectable joint difference |

Note. CA = Civic Actions; CI = Civic Intentions; HTMT = heterotrait–monotrait ratio. The combined evidence supports cautious separate reporting of CA and CI while precluding claims that the outcome-specific paths differ in magnitude.

**Table S8. Unmeasured latent method construct sensitivity analysis**

| **Structural path** | **Baseline MLR β** | **Equal-loading ULMC β** | **Absolute change** | **Conclusion** |
| --- | --- | --- | --- | --- |
| BLE → SLE | .530 | .530 | .000 | Stable |
| BLE → CA | .490 | .490 | .000 | Stable |
| BLE → CI | .447 | .447 | .000 | Stable |
| SLE → CA | .375 | .375 | .000 | Stable |
| SLE → CI | .396 | .396 | .000 | Stable |

Note. The equal-loading orthogonal method-factor model was admissible under MLR, Δχ²(1) ≈ 0, p = .999741, with mean standardized method variance of approximately 1.62 × 10⁻¹². Its structural paths and indirect associations were practically unchanged. The WLSMV equal-loading model produced a near-singular estimated-parameter covariance-matrix warning. A freely estimated method-loading MLR model was inadmissible because X1_TP_7 had a negative residual variance and was not interpreted. ULMC does not directly assess context-specific social desirability.

**Table S9. Exploratory fsQCA solutions under the large-sample specification**

| **Outcome** | **Directional intermediate solution** | **Consistency** | **PRI** | **Coverage** | **Multiplicity** |
| --- | --- | --- | --- | --- | --- |
| CA | TP*SP*BE*EE*CE | .908 | .826 | .411 | — |
| ~CA | ~TP*~SP*~EE + ~TP*~SP*~BE*~CE | .875 | .778 | .532 | 16 tied parsimonious models; first minimum displayed |
| CI | TP*CP*BE*EE*CE + TP*SP*BE*EE*CE | .891 | .797 | .439 | — |
| ~CI | ~TP*~SP*~BE*~EE*~CE | .919 | .830 | .396 | — |

Note. The specification used the stated composite scoring, direct 95/50/5 calibration, frequency threshold = 31, consistency threshold = .85, and PRI threshold = .75. The maximum necessity consistency was .756 under this specification and .827 across all scoring and calibration checks, below the .90 criterion. “~” denotes set negation; “*” denotes conjunction; “+” denotes alternative solution terms.

**Table S10. Stability of exploratory fsQCA solutions across 960 specifications**

| **Outcome** | **Specifications** | **Successful** | **No positive row** | **Computational failures** | **Unique printed expressions** | **Unique normalized expressions** | **Modal expression** | **Modal share** |
| --- | --- | --- | --- | --- | --- | --- | --- | --- |
| CA | 240 | 226 | 0 | 14 | 37 | 37 | TP*SP*BE*EE*CE | .177 |
| ~CA | 240 | 240 | 0 | 0 | 23 | 23 | ~TP*~SP*~BE*~EE*~CE | .100 |
| CI | 240 | 204 | 0 | 36 | 20 | 19 | TP*CP*BE*EE*CE + TP*SP*BE*EE*CE | .294 |
| ~CI | 240 | 180 | 60 | 0 | 7 | 7 | ~TP*~SP*~BE*~EE*~CE | .500 |

Note. The grid crossed prespecified scoring, calibration, frequency, consistency, and PRI choices, producing 240 specifications per outcome. The number and composition of solution expressions varied materially across settings.

**Table S11. Exploratory configuration unions compared with a five-condition majority rule**

| **Outcome** | **Large-sample solution** | **Direction-aligned majority rule** | **Pearson r** | **Crisp Jaccard** | **Majority-rule cases** |
| --- | --- | --- | --- | --- | --- |
| CA | TP*SP*BE*EE*CE | At least five high conditions | .777 | .604 | 596 |
| ~CA | ~TP*~SP*~EE + ~TP*~SP*~BE*~CE | At least five low conditions | .741 | .715 | 481 |
| CI | TP*CP*BE*EE*CE + TP*SP*BE*EE*CE | At least five high conditions | .801 | .705 | 596 |
| ~CI | ~TP*~SP*~BE*~EE*~CE | At least five low conditions | .759 | .520 | 481 |

Note. The configuration unions showed moderate-to-strong alignment with the simple rule that at least five of the six conditions were high or low. The comparison provides context for the incremental configurational information and does not imply that the two summaries are identical.

**Table S12. Exploratory fsQCA solutions with a frequency threshold of 1**

| **Outcome** | **Directional intermediate solution** | **Consistency** | **PRI** | **Coverage** |
| --- | --- | --- | --- | --- |
| CA | SP*CP*BE*EE*CE + TP*CP*BE*EE*CE + TP*SP*BE*EE*CE + TP*SP*CP*EE*CE + TP*SP*CP*BE*CE + TP*SP*CP*BE*EE | .866 | .768 | .564 |
| ~CA | ~TP*~SP*~BE*~EE + ~TP*~SP*~CP*~BE*~CE + ~TP*~SP*~CP*~EE*~CE + ~TP*~CP*~BE*~EE*~CE | .885 | .795 | .533 |
| CI | SP*CP*BE*EE*CE + TP*CP*BE*EE*CE + TP*SP*EE*CE + TP*SP*CP*BE*CE + TP*SP*CP*BE*EE | .861 | .756 | .555 |
| ~CI | ~TP*~SP*~CP*~BE*~EE + ~TP*~SP*~CP*~BE*~CE + ~TP*~SP*~CP*~EE*~CE + ~TP*~SP*~BE*~EE*~CE + ~TP*~CP*~BE*~EE*~CE | .869 | .757 | .530 |

Note. The specification used direct 95/50/5 calibration, frequency threshold = 1, consistency threshold = .80, and PRI threshold = .70. All 64 logical truth-table rows were represented, leaving no logical remainders for counterfactual simplification. Complex, parsimonious, and intermediate solutions therefore coincided.
